# Cost-effectiveness analysis of capecitabine monotherapy versus capecitabine plus oxaliplatin in elderly patients with advanced gastric cancer

**DOI:** 10.1371/journal.pone.0199553

**Published:** 2018-06-28

**Authors:** Jingyuan Peng, Chongqing Tan, Xiaohui Zeng, Shikun Liu

**Affiliations:** 1 Department of Pharmacy, The Third Xiangya Hospital, Central South University, Changsha, People’s Republic of China; 2 Department of Pharmacy, The Second Xiangya Hospital, Central South University, Changsha, People’s Republic of China; 3 PET-CT, The Second Xiangya Hospital, Central South University, Changsha, People’s Republic of China; 4 Center of Clinical Pharmacology, The Third Xiangya Hospital, Central South University, Changsha, People’s Republic of China; Universidade do Algarve Departamento de Ciencias Biomedicas e Medicina, PORTUGAL

## Abstract

**Background:**

There is no single standard chemotherapy regimen for elderly patients with advanced gastric cancer (AGC). A phase III trial has confirmed that both capecitabine monotherapy and capecitabine plus oxaliplatin are well tolerated for elderly patients with AGC, but their economic influence in China is unknown.

**Objective:**

The purpose of this cost-effectiveness analysis was to estimate the effects of capecitabine monotherapy and capecitabine plus oxaliplatin in elderly patients with AGC on health and economic outcomes in China.

**Methods:**

We created a Markov model based on data from a Korean clinical phase III trial to analyze the cost-effectiveness of the treatment of elderly patients in the capecitabine monotherapy (X) group and capecitabine plus oxaliplatin (XELOX) group. The costs were obtained from published reports and the local health system. The utilities were assumed on the basis of the published literature. Costs, quality-adjusted life years (QALYs), and incremental cost-effectiveness ratios (ICER) were estimated. One-way and probabilistic sensitivity analyses (Monte Carlo simulations) were performed.

**Results:**

In the cost-effectiveness analysis, X had a lower total cost ($45,731.68) and cost-effectiveness ratio ($65,918.93/QALY). The one-way sensitivity analysis suggested that the most influential parameter was the risk of requiring second-line chemotherapy in XELOX group. The probabilistic sensitivity analysis predicted that the X regimen was cost-effective 100% of the time, given a willingness-to-pay threshold of $26,598.

**Conclusions:**

Our findings show that the XELOX regimen is less cost-effective compared to the X regimen for elderly patients with AGC in China from a Chinese healthcare perspective.

## Introduction

Gastric cancer (GC) is the third leading cause of cancer-related deaths worldwide. Compared to more developed countries, GC is more common in less developed countries, and half of the world’s cases occur in Eastern Asia, mainly in China[1]. According to GLOBOCAN 2012, it was estimated that approximately 405,000 new cases of gastric cancer occured in China, which is the second most common malignancy, and 325,000 related deaths occured, which ranks third in cancer-related deaths in China[2]. Current epidemiologic data indicate that the incidence of GC increases progressively with age, and patients whose ages ranged from 50–70 be in the majority[3]. The therapeutic regimens for elderly patients are complex because of the physiological changes of the organ function and the increased prevalence of complications. Until now, no single standard chemotherapy regimen for patients with advanced gastric cancer (AGC) has been established worldwide, especially for elderly patients. According to the guidelines of the European Society for Medical Oncology (ESMO), GC treatment regimens for elderly patients that have been explicitly addressed in phase II trials with comparable survival results include capecitabine and oxaliplatin, FOLFOX (leucovorin, 5-FU and oxaliplatin), capecitabine and S1 (in Asian patients) [III,B][4]. Compared with some triple-drug schedules, such as DCF (docetaxel, cisplatin and 5-FU), which has been proved to be an effective regimen in advanced disease, the PF (cisplatin and 5-FU) combination seems to be more appropriate for elderly patients because of its lower toxicity[5]. However, PF also has some disadvantages, including its inconvenience and association with venous thrombosis and infections. On one hand, in order to reduce renal toxicity, cisplatin requires venous hydration; on the other hand, the continuous infusion of 5-FU requires using an indwelling catheter chronically.

Oxaliplatin is an alkylating agent that can inhibit the synthesis of DNA. Some studies have shown that oxaliplatin-based regimens are active and tolerable in elderly patients with AGC[6,7]. In addition, the incidence of nausea and renal toxicity relate to oxaliplatin is lower than that relate to cisplatin. Capecitabine is an oral fluoropyrimidine, which can be converted to 5-FU in tumor tissues with the action of the enzyme thymidine phosphorylase. Several studies have indicated that 5-FU can be substituted by capecitabine[8,9]. The XELOX (capecitabine plus oxaliplatin) regimen makes administration more convenient. It has already proven to be effective and is conveniently delivered as first-line treatment for patients with AGC[7,10–12].

Currently, there are few studies concerning the economic evaluation of the XELOX strategy in gastric cancer worldwide. Bin Wu et al.[13] compared the cost-effectiveness of the XELOX regimen and S-1 regimen as adjuvant chemotherapy after D2 gastrectomy for gastric cancer patients. Chongqing Tan et al.[14] analyzed the cost-effectiveness of XELOX regimen and S-1 as adjuvant chemotherapy after D2 gastrectomy and D2 gastrectomy alone for patients with stage II–IIIB gastric cancer.

Recently, a clinical phase III trial has confirmed that both capecitabine monotherapy and XELOX regimens are well tolerated for elderly patients with AGC, and the XELOX regimen is a feasible option[15], but economic evaluations of these treatments in China have not been performed. Hence, it is important to consider the value of the two regimens relative to their benefit and choose the preferable one for Chinese elderly patients.The purpose of this study was to compare the cost-effectiveness of two first-line chemotherapy regimens for elderly patients with AGC based on a Korean study from a Chinese healthcare perspective.

## Materials and methods

### Patients’ characteristics and treatments

Fifty patients aged 70 years or older with histologically confirmed, measurable AGC were enrolled in a clinical phase III trial in Korea (the ClinicalTrials.gov identifier: NCT01470742)[15]. The inclusion criteria included Eastern Cooperative Oncology Group (ECOG) performance status ≤two, life expectancy of at least three months and adequate bone marrow, hepatic and renal functions, no prior chemotherapy or only adjuvant chemotherapy that had been completed more than six months before registration and no radiotherapy within four weeks before registration, no any severe comorbid illness or a known history of anaphylaxis of any origin. In this study, patients were randomized into two groups: the X arm or XELOX arm. For the X arm (monotherapy regimen), capecitabine was administered at a dose of 1000 mg/m^2^ in 2 divided doses from day 1 to day 14; for the XELOX arm (combination regimen), oxaliplatin at a dose of 110 mg/m^2^ was given by intravenous injection on day 1, along with X. The two regimens were repeated every 21 days until disease progression, unacceptable toxicity, or the patients were requested to dropout. The patient characteristics for each arm were listed in Table 1.

**Table 1:** Patient characteristics for each arm.

	X arm (n = 26)	XELOX arm (n = 24)
**Median (range) age, yrs**	77 (70–83)	75 (70–84)
**Performance status**		
0–1	20	20
2	6	4
**Charlson’s comorbidity index**		
6	21	21
>6	5	3
**Previous gastrectomy**	15	11

### Clinical outcomes

In this Korean study cohort, the median PFS (progression-free survival) and OS (overall survival) of the two arms were significantly different. In the X group, the median PFS and OS were 2.6 months and 6.3 months, respectively, while those in the XELOX group were 7.1 months and 11.1 months, respectively. As the most common grade 3 or 4 adverse events (AEs) of chemotherapy, anemia, fatigue, anorexia diarrhea and Thrombocytopenia occurred in 15.4, 7.7, 7.7, 7.7 and 3.8% of patients, respectively, in the X arm, and 8.3, 12.5, 12.5, 4.2 and 4.2% of patients, respectively, in the XELOX arm. For the second-line treatment, 4 regimens were offered to 24 patients after failure of the first-line chemotherapy. They included ramucirumab with or without paclitaxel, irinotecan, oral tyrosine kinase inhibitors, and others. Table 2 shows the clinical outcomes for each arm.

**Table 2:** Clinical outcomes for each arm.

	X arm (n = 26)	XELOX arm (n = 24)
Risk of main AEs (grade 3 or 4), n(%)		
Anemia	4 (15.4%)	2 (8.3%)
Fatigue	2 (7.7%)	3 (12.5%)
Anorexia	2 (7.7%)	3 (12.5%)
Diarrhea	2 (7.7%)	1 (4.2%)
Thrombocytopenia	1 (3.8%)	1 (4.2%)
**Median PFS, months**	2.6	7.1
**Median OS, months**	6.3	11.1

### The strategies and Markov model structure

**Fig 1:**
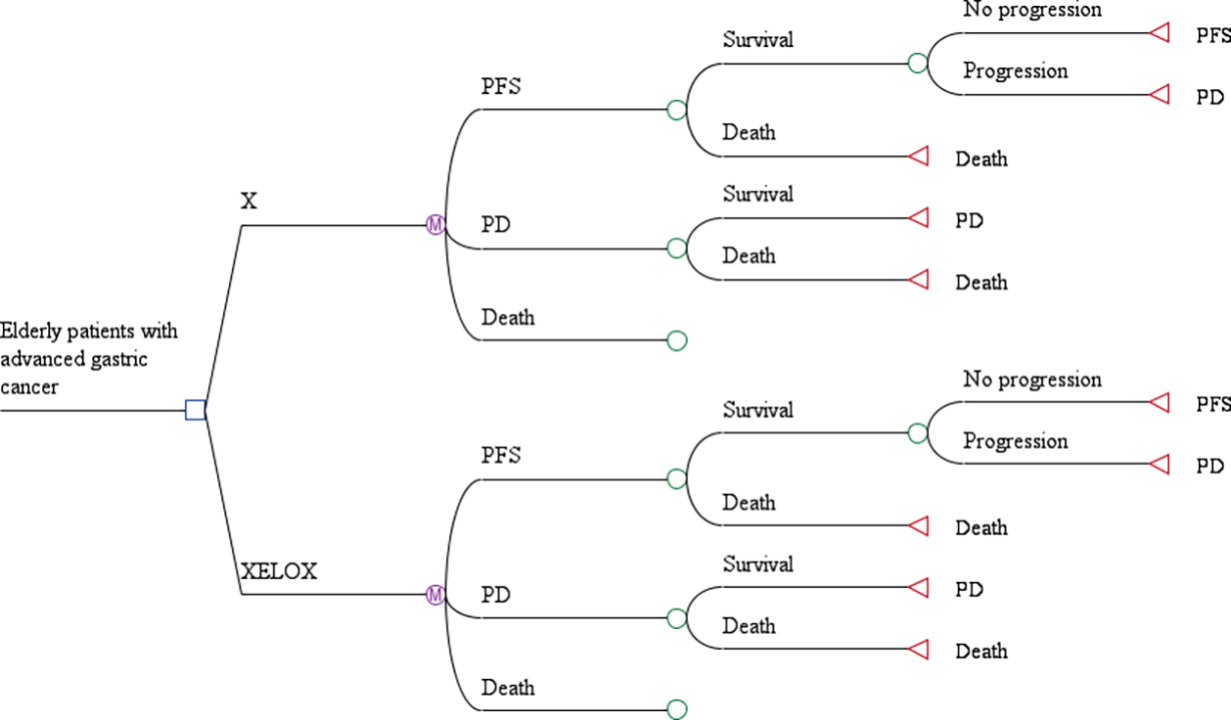
Markov model for elderly patients with advanced gastric cancer.

A Markov model comprising 3 mutually exclusive health states was constructed: progression-free survival (PFS), progressive disease (PD) and death (Fig 1). Patients who received either the X treatment or XELOX therapy as the first-line treatment were assumed to begin their treatment at the PFS state in our model. In the PD state, patients received 1 of 4 second-line treatments. The transition probabilities were obtained from the Kaplan-Meier curves in the trial. We used GetData Graph Digitizer software to extract the data from the Kaplan-Meier curves and simulated Log-logistic survival models using R software. Meanwhile, we examined the goodness of fit of several possible distributions using AIC (Akaike Information Criterion) and BIC (Bayesian Information Criterions) as evaluation indexes. The estimated κ and θ parameters and adjusted R^2^ values are listed in Table 3. The discount rate was set at 3%.

**Table 3:** Log-logistic parameters for progression-free survival (PFS) and overall survival (OS) for the two regimens.

	Arms	θ	κ	Adjusted R^2^
**OS**	X	-3.2726	1.4703	0.9366739
	XELOX	-4.5714	1.4960	0.9835455
**PFS**	X	-3.0531	2.1872	0.9949386
	XELOX	-3.9836	1.6494	0.9848411

### Measurement of costs

Costs were calculated from the Chinese healthcare perspective in 2016 US$. Direct medical costs mainly included costs of first-line treatments, second-line therapies, follow-up, grade 3 or 4 adverse event-related costs and other costs during the treatments (Table 4). The costs of administration, supportive care, main AEs (diarrhea, anemia, fatigue, anorexia and thrombocytopenia) were based on the literature by Wu[16,17]. The costs of capecitabin, oxaliplatin, abdominal CT, abdominal MRI, chest radiography and laboratory evaluations were obtained from the local medical system[18]. Detailed follow-up data were not referenced in the original study, so we referred to a previously published study on gastric cancer[14]. Detailed data about the main AEs were derived from the original study. Second-line chemotherapies included ramucirumab with or without paclitaxel, irinotecan, oral tyrosine kinase inhibitors, and others. In the original literature, specific regimens were not given, so we consulted the 2016 Chinese National Comprehensive Cancer Network (NCCN) Clinical Practice Guidelines in Oncology-Gastric Cancer and other articles[19–21] to calculate the costs.

**Table 4:** Baseline costs, utility values and risks in the two arms for elderly patients with advanced gastric cancer in China.

Parameters	Median	Range	Distribution
**Costs, $**			
Capecitabine per 500 mg[18]	4.8	3.8–5.8^a^	Lognormal
Oxaliplatin per 50 mg[18]	371.9	297.5–446.3^a^	Lognormal
Laboratory evaluations per unit	129.5	103.6–155.4^a^	Lognormal
Administration per unit[16]	18.5	16.6–20.3^c^	Lognormal
Supportive care per unit[17]	1415.4	1022.8–2021.5	Lognormal
Second-line chemotherapy per unit	5300.4	4240.3–6360.5^a^	Lognormal
Abdominal CT per unit	60.2	30.1–90.3^b^	Gamma
Abdominal MRI per unit	123.3	61.7–185.0^b^	Gamma
Chest radiograph per unit	6.0	3.0–9.0^b^	Gamma
**Expenditures on main AEs (grade 3 or 4), $[16]**			
Diarrhea per episode	44.3	39.9–48.7^c^	Lognormal
Anemia per episode	531.7	478.5–584.9^c^	Lognormal
Fatigue per episode	115.4	103.8–126.9^c^	Lognormal
Anorexia per episode	115.4	103.8–126.9^c^	Lognormal
Thrombocytopenia per episode	3551.7	3196.5–3906.9^c^	Lognormal
**Risk of main AEs in X (grade 3 or 4)[15]**			
Diarrhea	0.08	0.064–0.096^a^	Beta
Anemia	0.15	0.12–0.18^a^	Beta
Fatigue	0.08	0.064–0.096^a^	Beta
Anorexia	0.08	0.064–0.096^a^	Beta
Thrombocytopenia	0.04	0.032–0.048^a^	Beta
**Risk of main AEs in XELOX (grade 3 or 4)[15]**			
Diarrhea	0.04	0.032–0.048^a^	Beta
Anemia	0.08	0.064–0.096^a^	Beta
Fatigue	0.13	0.104–0.156^a^	Beta
Anorexia	0.13	0.104–0.156^a^	Beta
Thrombocytopenia	0.04	0.032–0.048^a^	Beta
**Risk of requiring second-line chemotherapy[15]**			
X	0.38	0.304–0.456^a^	Beta
XELOX	0.58	0.464–0.696^a^	Beta
**Utility[22]**			
PFS in two arms	0.797	0.638–0.956^a^	Beta
PD in two arms	0.577	0.462–0.692^a^	Beta

CT = computed tomography; MRI = magnetic resonance imaging; X = capecitabine; XELOX = capecitabine and oxaliplatin.

^a^Varied by ± 20%.

^b^Varied by ± 50%.

^c^Varied by ± 10%.

### Utility estimates

The utility estimates identified and used in the model were derived from the literature by Shiroiwa T et al[22]. They assessed quality of life by using the EuroQoL (EQ-5D) responses of the ToGA trial calculated by Japanese scoring algorithm. Because the EQ-5D was not administered after disease progression in the TOGA trial, they assumed the utility value of PD state based on National Institute for Health and Clinical Excellence (NICE) technology appraisal 208 (NICE, 2010). In our study, the values were set at 0.797 for the PFS state, 0.577 for the PD state and 0 for the death state in both groups.

### Sensitivity analysis

One-way sensitivity analysis was conducted to examine the impact of 29 variables defined in our model. To assess the influence of parameter uncertainties, we used a second-order Monte Carlo simulation with 1000 runs to perform a probabilistic sensitivity analysis. According to the Chinses Guidelines for Pharmacoeconomic Evaluations, the WTP threshold was set to $26,598, which was three times the per capita GDP of China in 2016.

## Results

A total of 99.26% of patients in the X arm and 97.70% in the XELOX arm had died when the model was used to track 15 years.

### Costs

In total, the incremental cost was $71,612.32 for the XELOX arm compared with the X arm. The cost of the X regimen was $45,731.68 and the cost of the XELOX regimen was $117,344.00. Detailed information is illustrated in Table 5.

**Table 5:** The base-case results of cost-effectiveness analysis.

Outcome	X	XELOX
**Costs ($)**		
Costs in PFS	3100.32	24533.40
Costs in PD	42631.36	92810.60
Total costs	45731.68	117344.00
Incremental costs	-	71612.32
**Effectiveness (QALYs)**		
QALYs in PFS	0.22	0.56
QALYs in PD	0.47	0.83
Total effectiveness	0.69	1.39
Incremental effectiveness	-	0.70
**Cost/Effectiveness**	65918.93	84114.04
**Incremental cost/effectiveness**	-	102113.38

### Effectiveness

In total, the incremental effectiveness was 0.70 QALYs for the XELOX arm compared with the X arm (1.39 QALYs versus 0.69 QALYs). In detail, for the PFS state, the effectiveness was 0.22 QALYs in the X arm and 0.56 QALYs in the XELOX arm; for the PD state, the effectiveness was 0.47 QALYs for the X arm and 0.83 QALYs for the XELOX arm (Table 5).

### Cost-effectiveness analysis

Obviously, the XELOX strategy was less cost-effective compared with the X group. The XELOX arm spent $84,114.04 per QALY compared with $65,918.93 per QALY for the X arm. The ICER for XELOX arm relative to X arm was $102113.38/QALY (Table 5).

### Sensitivity analysis

**Fig 2:**
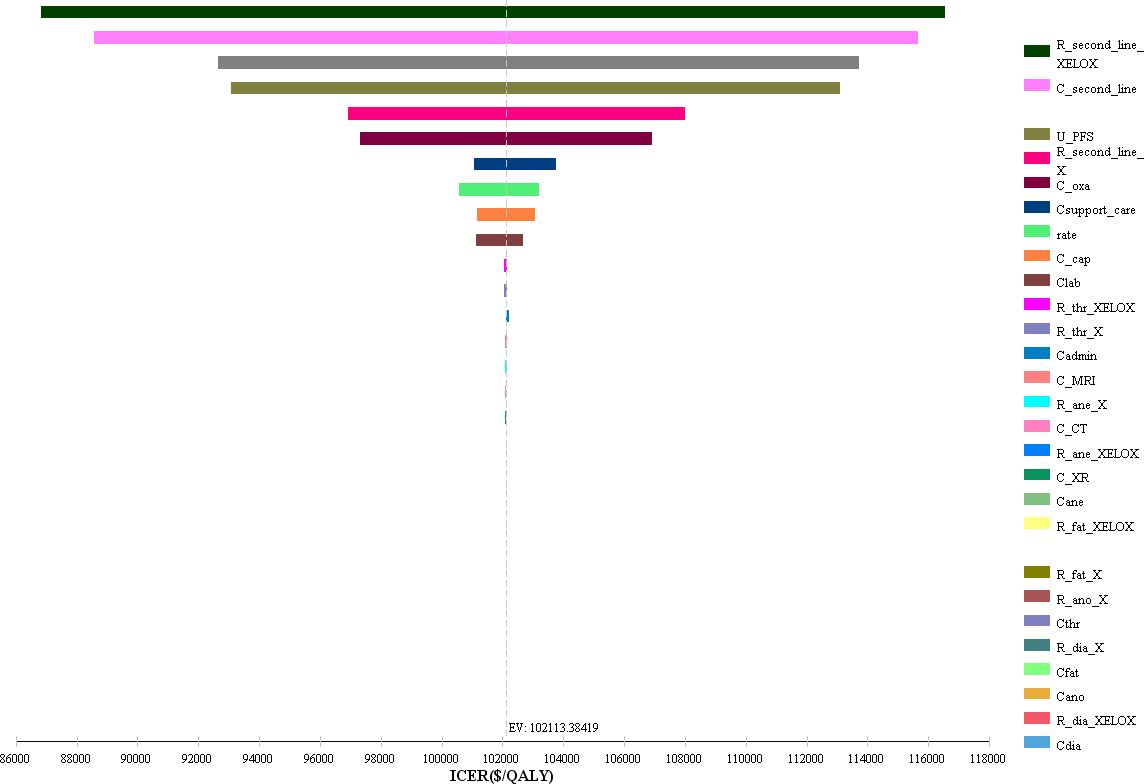
Tornado diagram for one-way sensitivity analysis.

In our model, three variables (the costs of abdominal CT, abdominal MRI and chest radiography) varied at a range of ±50%, the costs of administration and expenditures on main AEs varied at a range of ±10%. Other variables varied at a range of ±20%. The tornado diagram (Fig 2) shows the results of one-way sensitivity analysis, the risk of requiring second-line chemotherapy in XELOX group was the most influential parameter. When this parameter ranges from 0.464 to 0.696, the ICER increases from $86,812.291 per QALY to $116,559.667 per QALY. In addition, the cost of second-line treatment, the utility for PFS state, the risk of requiring second-line chemotherapy in X group and the cost of oxaliplatin were important influential factors for ICER.

### Probabilistic sensitivity analysis

**Fig 3:**
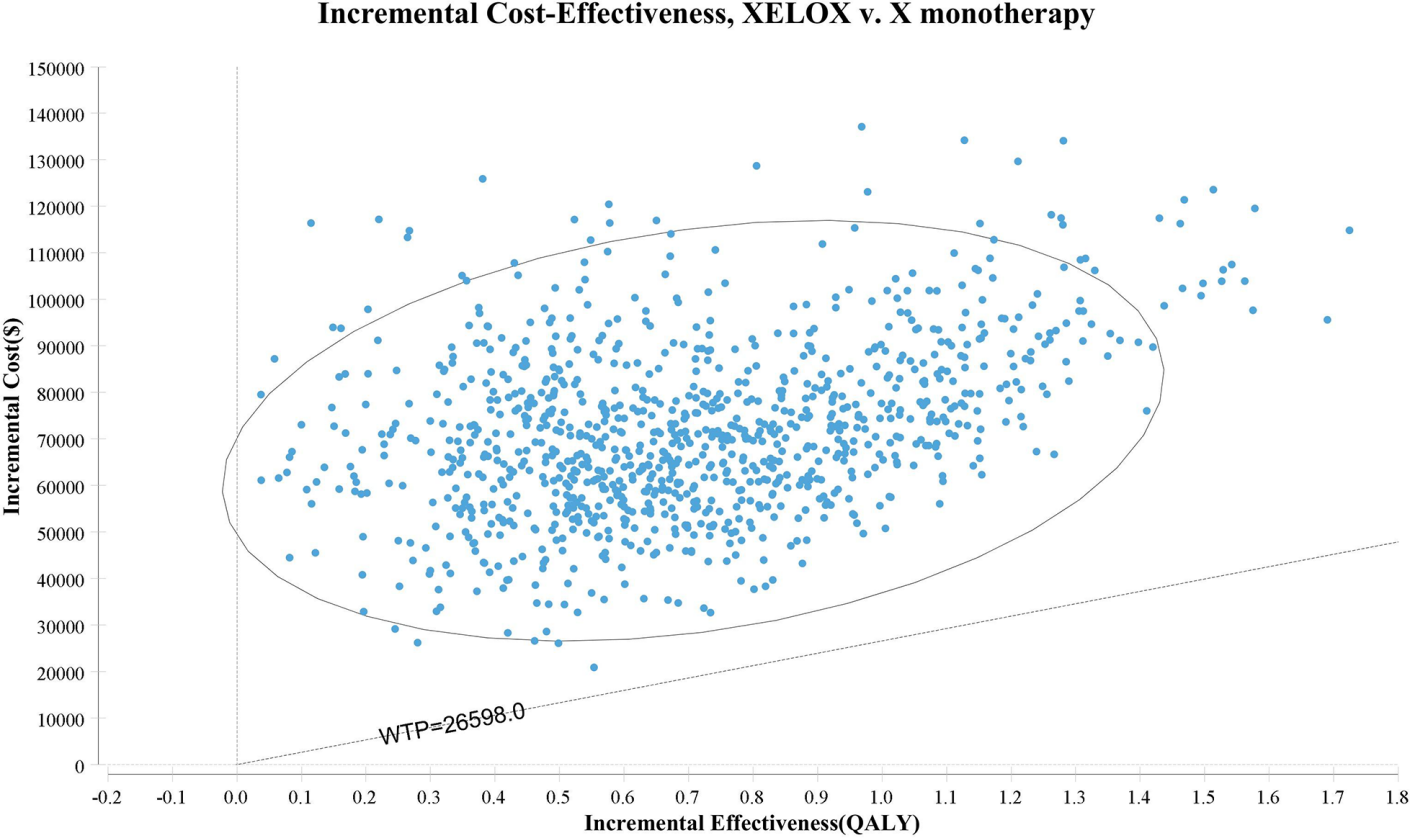
The scatter plot of Monte Carlo probabilistic sensitivity analysis for the X vs. XELOX strategies.

**Fig 4:**
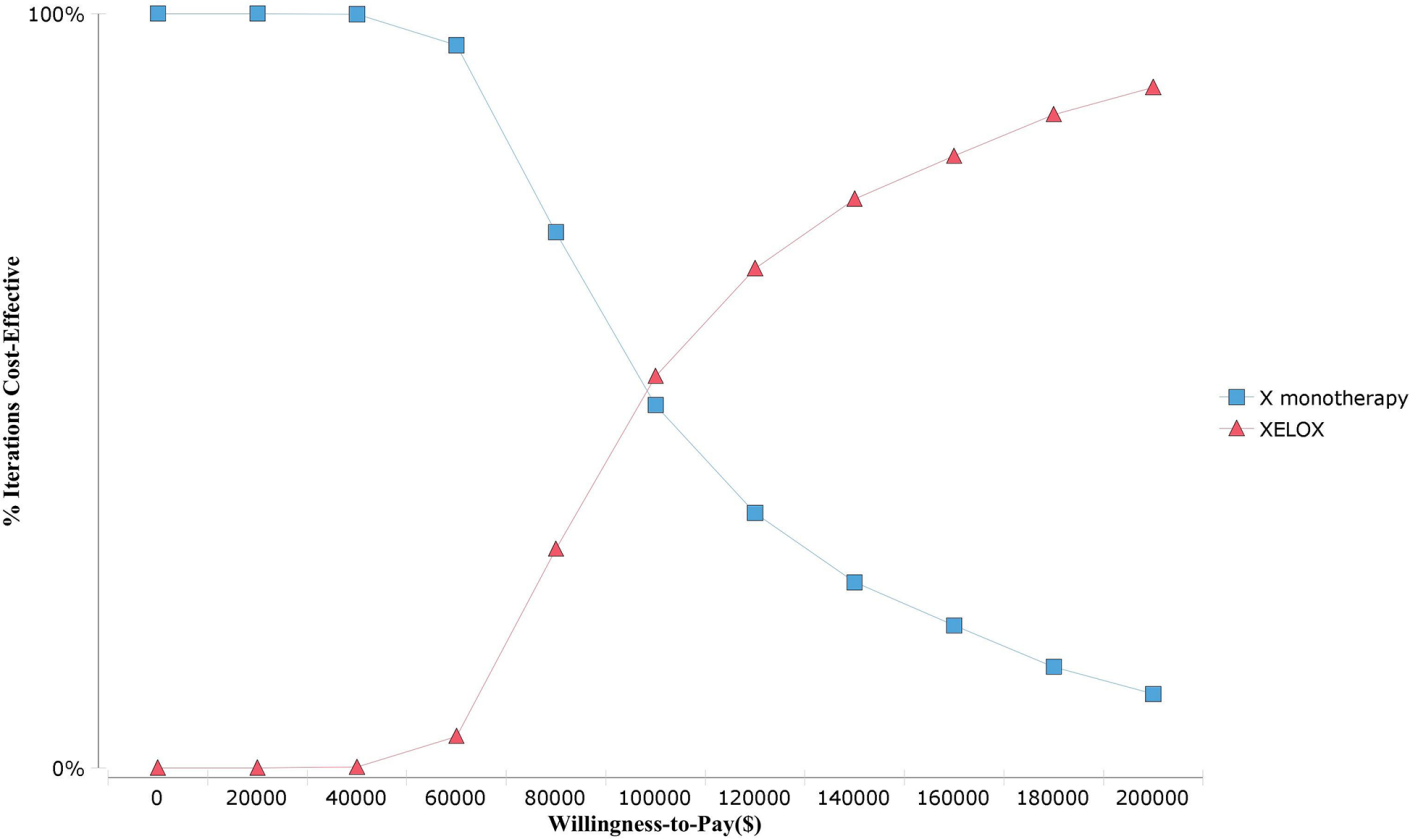
Cost-effectiveness acceptability curves. Different choices for elderly Chinese patients at various WTP thresholds.

The scatter plot of the Monte Carlo probabilistic sensitivity analysis for the X vs. XELOX (Fig 3) showed that compared to the XELOX strategy, the probability that the X strategy was cost-effective was 100%. The cost-effectiveness acceptability curves showed the probability of strategies in different WTP thresholds. Strategies with greater probability would be more cost-effective. As shown in Fig 4, the curve intersection was approximately $100,000. When the WTP value was $100,000, the acceptability of the X strategy and XELOX strategy were 0.48 and 0.52, respectively. When the WTP thresholds varied from $0 to $100,000, the X regimen may have been more acceptable, while as the WTP thresholds varied from $100,000 to $200,000, the XELOX regimen was considered to be optimal.

## Discussion

In this study, the cost-effectiveness of XELOX and X chemotherapy regimens for elderly patients with AGC was evaluated from a Chinese healthcare perspective. In brief, the Chinese healthcare system is financed by three main parties: Government, enterprise and individuals. The costs to government are principally covered by taxation and various sorts of user fees. Income tax, sales tax and turnover tax on enterprise are the main source of tax revenues in China. In addition, some enterprises (e.g. producer of targeted agent) often provide Chinese patients with the patients assistant program. However, individual payments also be of great importance in the current Chinese healthcare system[23]. Formulary is an acknowledged management of drugs for health insurance in China. Drugs listed in the formulary should meet some criteria, including being clinically needed, effective, safe, convenient to use, and reasonable price and sufficiency of market supply. The National Basic Medical Insurance Drug Formulary List, which is a primary reimbursement formulary in China, is codeveloped by the Ministry of Human Resources and Social Security and other related ministries[24]. In recent years, the Chinese government has showed an interest in Heath technology assessment (HTA) policy because of the increase of health care costs. Although the China Guidelines for Pharmacoeconomic Evaluations had been published in 2011, HTA is currently not implemented nationwide in China[25].

This study is the first to analyze the economic evaluation of XELOX and X chemotherapy regimens for elderly patients with AGC in China. Elderly patients have often been under-represented in clinical trials. Therefore, there are few economic evaluations concern elderly patients. Compared to previous study, a significant difference of our study is that we took elderly patients with AGC as the study subject. According to our analysis, although the XELOX arm gained more QALYs than the X arm (1.39 QALYs versus 0.69 QALYs), different costs in all aspects resulted in higher lifetime costs for the XELOX compared with the X arm. This may be mainly because the price of oxaliplatin is far higher than that of capecitabine.

In terms of cost-effectiveness acceptability, as seen from Fig 4, the more cost-effective regimen changed as the WTP threshold varied. In this study, the WTP threshold was set to $26598 and it was related to the Chinese per capita GDP which was an average value. In fact, the difference in the per capita GDP of different provinces in the Chinese mainland is obvious. The province with the highest per capita GDP is Tianjin, and the province with the lowest per capita GDP is Gansu. Their per capita GDPs were $17,406 and $4,141, respectively[26,27]. For all provinces, three times the per capita GDP was less than $100,000. Thus, the X regimen is a more cost-effective first-line chemotherapy than the XELOX regimen for elderly patients with AGC on the Chinese mainland.

Our tornado diagram showed that the risk of requiring second-line chemotherapy in XELOX group would be the most influential parameter that could reduce the ICER and the second most influential parameter was the cost of second-line treatment. Reducing the risk of requiring second-line chemotherapy in XELOX group and decreasing the cost of second-line treatment would be good ways to reduce the ICER. Next, the latter was mainly be discussed in this paper. In our study, there were four second-line chemotherapy regimens, and we calculated their costs by the proportional weight method. When we only chose the least expensive regimen (irinotecan), the ICER decreased to $53,148.44 per QALY gained, which was nearly half of $102,113.38, and the total costs of the XELOX arm decreased to $62,394.77. Although the X strategy would still be dominant when the WTP threshold was set to three times the Chinese per capita GDP, the XELOX regimen may be dominant in some provinces with a high per capita GDP. In addition, the cost of supportive care may also be an important parameter in our results.

In many published studies on the economic evaluation of the XELOX regimen, the results show that the XELOX strategy is a preferable regimen. Chongqing Tan et al.[14] determined that compared with S-1 and SO (surgery only), XELOX regimen was the best option as a first-line adjuvant chemotherapy after a D2 gastrectomy for patients with resectable gastric cancer in China. According to their survey, the total costs of the XELOX strategy was $44,658 when the model tracked data for 30 years. However, in our survey, when the model tracked data for 15 years, the evaluation of the XELOX arm reached up to $117,344.00, which was far higher than the results of Chongqing Tan et al. This was possible due to the difference in the patient characteristics of two studies. The subjects of our study were elderly patients whose ages ranged from 70–84, but the age range of their study was simply 18 years or older. In addition, all the patients in their study had D2 surgery and performed R0 resection, but some patients in our study underwent gastrectomy. Another possible reason was the difference in the second-line chemotherapy regimen, which caused the cost of second-line chemotherapy in our study to be higher than theirs. In terms of QALYs, the total QALYs in our study and theirs were 1.39 and 6.07, respectively.

Bin Wu et al.[13] also suggested that adjuvant therapy with XELOX regimen was a more favorable treatment option than the S-1 strategy. As reported by Bin Wu et al, the total costs of the XELOX regimen was $20,331.6 which was far lower than our evaluation ($117,344.00). This may be caused by the following reasons. Firstly, the cost of oxaliplatin was different. The cost of brand-name oxaliplatin (Eloxatin; Sanofi-Aventis, Hangzhou, China) which was used in our study is four times higher than that of generic oxaliplatin which was used in their study. Secondly, paclitaxel (80 mg/m^2^) was administered weekly for 3 of every 4 weeks as a second-line chemotherapy in their study. However, our study included 4 Second-line chemotherapies. At last, the difference in the patient characteristics was also an important influential factor.

We must consider several limitations in our research. First, our analysis was based on the information of a Korean phase III trial, which was not patient-level data. Second, although QOL was assessed by using the European Organization for Research and Treatment of Cancer questionnaire (EORTC QLQ-C30) in validated Korean version in the trial, it is difficult to convert a score to a utility. Thus, the utility used in our model was derived from published research. Because of the difference in two trials, the utility values which we used couldn’t completely suit the patients in our study. However, we conducted a sensitivity analysis to assess the influence on our results caused by these factors. Third, some of the costs were referenced to previously published articles. However, in further sensitivity analyses, the impact of varying these variables was small. At last, this study was based on a phase III trial in Korea that had a small sample size; only 50 patients were enrolled. This may have had an effect on the outcome.

In conclusion, our study demonstrated that the XELOX regimen presented more QALYs in the first-line treatment for AGC in China. However, based on our model, the XELOX strategy is likely not a cost-effective choice for elderly patients with AGC in the Chinese mainland when compared to the X strategy, from a Chinese healthcare perspective. In addition, the government could make elderly patients enjoy more preferential policies to satisfy their demand for treatment in China.

## Supporting information

S1 Dataset Relevant data in the Markov model. (XLS)
